# Assessment of mosaic loss of chromosome Y in pulmonary fibrosis reveals limited association with susceptibility or disease severity

**DOI:** 10.1136/bmjresp-2025-003846

**Published:** 2026-02-10

**Authors:** Dapeng Wang, Niran Hadad, Samuel Moss, Elena Lopez-Jimenez, Simon R Johnson, Toby M Maher, Philip L Molyneaux, Yajie Zhao, John R B Perry, Paul J Wolters, Jonathan A Kropski, R Gisli Jenkins, Nicholas E Banovich, Iain Stewart

**Affiliations:** 1 National Heart and Lung Institute, Imperial College London, London, UK; 2 Shandong Key Laboratory of Intelligent Oil & Gas Industrial Software, Qingdao Institute of Software, College of Computer Science and Technology, China University of Petroleum (East China), Qingdao, China; 3 Translational Genomics Research Institute, Phoenix, Arizona, USA; 4 Centre for Respiratory Research, NIHR Biomedical Research Centre and Biodiscovery Institute, Translational Medical Sciences, University of Nottingham, Nottingham, UK; 5 Keck Medicine of University of Southern California, Los Angeles, California, USA; 6 MRC Epidemiology Unit, Wellcome-MRC Institute of Metabolic Science, University of Cambridge School of Clinical Medicine, Cambridge, UK; 7 Department of Medicine, University of California, San Francisco, California, USA; 8 Division of Allergy, Pulmonary and Critical Care Medicine, Department of Medicine, Vanderbilt University Medical Center, Nashville, Tennessee, USA; 9 Department of Cell and Developmental Biology, Vanderbilt University, Nashville, Tennessee, USA; 10 Department of Veterans Affairs Medical Center, Nashville, Tennessee, USA

**Keywords:** Idiopathic Pulmonary Fibrosis

## Abstract

**Background:**

Pulmonary fibrosis (PF) is a rare lung disease with diverse pathogenesis and biological mechanisms. Mosaic loss of chromosome Y (mLOY) has been reported to be associated with increased risk of fibrotic diseases. However, the exact role of mLOY in the development of PF remains to be elucidated.

**Methods:**

Copy number on chromosome Y was used to estimate mLOY comparing patients in PROFILE and gnomAD cohorts and between cases and control patients from the GE100KGP cohort. Correlation of mLOY with demographic and clinical variables was tested using patients from the PROFILE cohort. Lung single-cell transcriptomic data were analysed to assess the cell types implicated in mLOY. Mendelian randomisation was performed to examine the causal relationship between mLOY, idiopathic pulmonary fibrosis (IPF) and telomere length.

**Results:**

The genetic analysis suggests that mLOY is found in PF from both case cohorts but when compared with an age matched population the effect is minimal (p=0.00316, median: 0.288 vs 0.291). mLOY is related to age (p=0.000214) and shorter telomere length (p=0.00815) rather than PF severity or progression. Single-cell analysis indicates that mLOY appears to be found primarily in immune cells. Mendelian randomisation demonstrates that mLOY is not on the causal pathway for IPF, but partial evidence supports that telomere shortening is on the causal pathway for mLOY.

**Conclusions:**

Our study confirms the existence of mLOY in PF patients, suggests that mLOY is not a major driver of IPF, and might support a triangulation model where telomere shortening leads to both IPF and mLOY.

**WHAT IS ALREADY KNOWN ON THIS TOPIC**

- Mosaic loss of chromosome Y (mLOY) is linked to ageing and fibrotic diseases.

**WHAT THIS STUDY ADDS**

- mLOY is present in pulmonary fibrosis (PF) patients, primarily in immune cells, and is associated with age and shorter telomeres, but not with disease severity or progression. Mendelian randomisation suggests mLOY is not a causal driver of idiopathic PF (IPF), while telomere shortening may be upstream of both mLOY and disease.

**HOW THIS STUDY MIGHT AFFECT RESEARCH, PRACTICE OR POLICY**

- Telomere shortening emerges as a key factor in IPF, and mLOY may serve as a biomarker to stratify patients or guide mechanistic studies rather than a direct therapeutic target.

## Introduction

 Pulmonary fibrosis (PF) is a chronic progressive lung disease associated with ageing.^1^ PF has a very poor prognosis with limited treatment options.^2^ The pathogenesis of PF is thought to involve repeated epithelial injury leading to fibroblast activation and abnormal extracellular matrix deposition.^3^ Large-scale population-based genetic analysis and genome-wide association studies (GWAS) have identified several genes that play crucial regulatory roles in spindle assembly checkpoint (SAC) and proper chromosome segregation, such as *SPDL1*,^4^ *KIF15*,^5^ *MAD1L1*^5^ and *KNL1*.^6^ Impairment and disruption of SAC mechanism could lead to ploidy errors and aneuploidy.^7^

Mosaic loss of chromosome Y (mLOY) is the most common form of aneuploidy relating to genome instability in non-malignant diseases and can occur in all age groups but is most prevalent in older males^8^ and has been associated with smoking.^9^ GWAS variants significantly associated with mLOY are enriched among genes implicated in cell cycle regulation, haematologic malignancies and cancer susceptibility, suggesting the shared genetic architecture between mLOY and cancer susceptibility.^10^,^12^

It is reported that mLOY in blood is associated with cardiac dysfunction and fibrotic phenotype in various organs in a murine model,^13^ and mLOY in blood cells profoundly contributes to the formation of cardiac fibrosis and the high mortality of patients after transcatheter aortic valve replacement via the activation of TGF-β signalling pathway.^14^ Although there is some evidence that mLOY might contribute to fibrogenesis, it is unclear whether mLOY is directly causally associated with PF or its severity. We, therefore, hypothesised that the genetic variants associated with idiopathic pulmonary fibrosis (IPF) would promote fibrogenesis via mLOY. To test this hypothesis, we assessed the copy number on chromosome Y in two case-control cohorts and the correlation between mLOY and demographic/clinical variables in the PROFILE cohort. Second, we examined cell types that could be driving the difference between disease and control using single-cell transcriptomic data. Thirdly, we conducted Mendelian randomisation between mLOY, IPF and telomere length.

## Methods

### Cohorts

The whole genome sequencing data from two independent PF case cohorts were used in the study: PROFILE cohort and GE100KGP cohort. PROFILE is a UK-based cohort of treatment-naive PF patients recruited from two locations including London and Nottingham in the UK.^4^ This cohort comes with a rich set of demographic and clinical baseline and longitudinal meta data.^15^ GE100KGP case cohort is defined based on the health record data and/or clinically diagnosed familial pulmonary fibrosis (FPF) diseases from the 100K Genome Project run and managed by Genomics England, which has been described previously.^16^ For each cohort, only male patients of European ancestry were used for the analysis of mLOY. Sample metadata for samples in the HGDP and 1 KG subsets were downloaded from gnomAD (V.3.1.2) as controls for PROFILE.^17^ To mitigate the confounding effect of age, nearest neighbour propensity score matching was performed to generate the non-PF controls in the rare disease programme from 100K Genome Project data to match the case cohort based on age information using the MatchIt package.^18^

### Estimate of copy number

The male-specific region Y (MSY) is unique to chromosome Y and not shared by chromosome X. Therefore, MSY was chosen for analysis. The copy number of chromosome Y was defined as the mean read depth on MSY divided by mean read depth on 22 autosomes, unless otherwise stated. The read depth was calculated using SAMtools from the BAM files of whole genome sequencing data mapped onto human reference genome (GRCh38).^19^ Due to data availability, a different approach was used to measure copy number on chromosome Y for samples from the gnomAD cohort: copy number on chromosome Y was defined as the sample’s mean depth on MSY divided by sample’s mean depth across chromosome 20. Patients in the PROFILE cohort were divided into the mLOY group and the non-mLOY group, where the mLOY group is defined as those patients with less than mean minus SD of copy number on chromosome Y to ensure sufficient statistical power.

### Correlation analysis with clinical and demographic variables in PROFILE cohort

Five continuous variables including age, per cent of predicted forced vital capacity (ppFVC), per cent of predicted transfer factor of the lung for carbon monoxide (ppTLCO), composite physiologic index (CPI), telomere length and two discrete variables including Gender-Age-Physiology (GAP) stage and disease progression in 12 months were selected to study their correlation with mLOY where disease progression was defined as a 10% relative decline in FVC or death within 12 months from the baseline visit. Case-by-case adjudication of disease progression was performed by clinical teams where 12-month lung function data were missing. Fisher’s exact test was used for testing the relationship between mLOY and risk alleles from four SAC genes. The Wilcoxon test and Kruskal-Wallis test were used for testing between two groups and three groups, respectively. Spearman’s rank correlation coefficient was used for the correlation analysis between two variables. The significance of the tests was determined at p<0.05.

### Single-cell analysis

Single-cell analysis was carried out using 108 samples collected from 71 male patients consisting of 27 controls and 44 PF cases, including 26 IPF, 6 interstitial lung disease (ILD), 1 connective tissue disease-associated ILD (CTD-ILD), 3 coal worker’s pneumoconiosis (CWP), 2 sarcoidosis, 2 non-specific interstitial pneumonia (NSIP), 2 interstitial pneumonia with autoimmune features and 2 chronic hypersensitivity pneumonitis (cHP) cases from a previously published single-cell RNA-sequencing dataset (GSE227136).^20^ Data were collapsed in cases where multiple samples were collected from the same patient, intended to represent less and more fibrotic regions, unless specified otherwise. CellRanger output BAM files were used to extract reads uniquely mapped to chromosome Y and autosomes. Per cell, mLOY was determined as the ratio between reads mapped to chromosome Y and reads mapped to all autosomes, limiting the analysis to cells that passed filtering using the filtering criteria described in.^20^ Cells with a chrY/autosomes ratio equal to 0 were considered cells with mLOY. The proportion of mLOY for a sample was determined as the proportions of cells without reads mapped to chromosome Y (mLOY=0).

To assess the probability of mLOY between disease and control, and with disease progression (less fibrotic compared with more fibrotic samples), cells were assigned to a binary mLOY positive or mLOY negative category and a logistic regression model was fitted for each of the following covariates: disease type, lineage and cell identity. Significance was set to false discovery rate (FDR)-adjusted p<0.05.

Differential expression was assessed between mLOY cells and the top 25th percentile of non-mLOY cells in disease samples using negative binomial mixed models in both cell-agnostic and cell-type aware manner. A model was fitted for each cell type assessing changes to gene expression associated with mLOY using the *glmmTMB* package^21^ with a negative binomial distribution and a log link function. Sample identity was used as a random effect to account for dispersion associated with single-cell data. Y-chromosome genes were excluded from the analysis, and genes with FDR-adjusted p<0.05 were considered as significant. Standardised regression coefficients were obtained using the *effectsize* package^22^ and used as input for gene set enrichment analysis (GSEA) using the *clusterProfiler* package.^23^

### Mendelian randomisation

This two-sample MR approach used public GWAS summary statistics to identify genetic instruments (single nucleotide polymorphisms; SNPs) for IPF susceptibility (4125 cases, 20 464 controls),^6^ ovarian cancer (25 509 cases, 40 941 controls),^24^ prostate cancer (79 148 cases, 61 106 controls)^25^ and telomere length (leucocyte) (sample size: 472 174).^26^ For the mLOY estimation, the same approach as described by Liu *et al*^27^ was used for mLOX estimation to get an enhanced mLOY calling (sample size: 204 770). GWAS was performed based on the enhanced mLOY calling using BOLT-LMM, which was the same as the GWAS for PAR-LOY.^10^ All studies used for instrument selection were performed using European ancestry only, or majority European ancestry, cohorts to minimise possible violation of the independence assumption due to population stratification.

Genetic instruments were filtered for genome-wide significance (p<5×10^−8^) and clumped to ensure independence using the 1000 Genomes Project reference panel (r2<0.001 within a 10 000 kb window). All SNPs were required to be strong instruments for each exposure (F>10) to avoid weak instrument bias. These steps aimed to ensure that selected instruments were reliably associated with the exposure to avoid violation of the relevance assumption.

A primary bidirectional MR analysis was performed to assess for causal effects between mLOY and IPF. A random-effects inverse-variance weighted (IVW-RE) MR method^28^ was used to provide main causal estimates (Mendelian Randomisation).^29^ MR-Egger^30^ and weighted median^31^ methods were used to provide pleiotropy-robust MR estimates. MR-PRESSO^32^ and MR-Lasso^33^ methods were used to provide pleiotropy-robust MR estimates by identifying and removing genetic instruments with likely horizontal pleiotropic effects. The use of multiple pleiotropy-robust methods takes advantage of the different assumptions underlying each approach to provide more reliable outcomes.^34^ Outcomes from MR analyses were therefore considered to be robust if causal estimates were consistent across all five methods. This use of multiple complementary methods also aimed to minimise the risk of false positive outcomes that may be inadvertently confounded through violation of the exclusion restriction assumption. Leave-one-out analyses were performed to identify SNPs that are independently driving or distorting causal effect estimates. As recommended by the current methodological guidance in MR analyses, a positive control analysis should reflect an established causal relationship, while negative control analyses involve testing for a causal effect between the selected genetic instrument for an exposure and an outcome that should not be causally linked, aiming to highlight potential violation of the instrumental variable assumptions if deviation from the expected effects is observed.^35^ A positive control analysis assessed the effects of the mLOY instrument on prostate cancer risk. As mLOY is a sex-specific trait, a negative control analysis assessing effects of the mLOY instrument on ovarian cancer risk was also performed. As shortened telomere length is a risk factor for IPF, a secondary analysis was performed to assess if telomere length may also have causal effects on mLOY. All MR analyses were performed in R (V.4.2.2).

### Patient and public involvement

Patients or the public were not involved in the design, or conduct, or reporting, or dissemination plans of our research.

## Results

### mLOY is observed in PF

**Figure 1:**
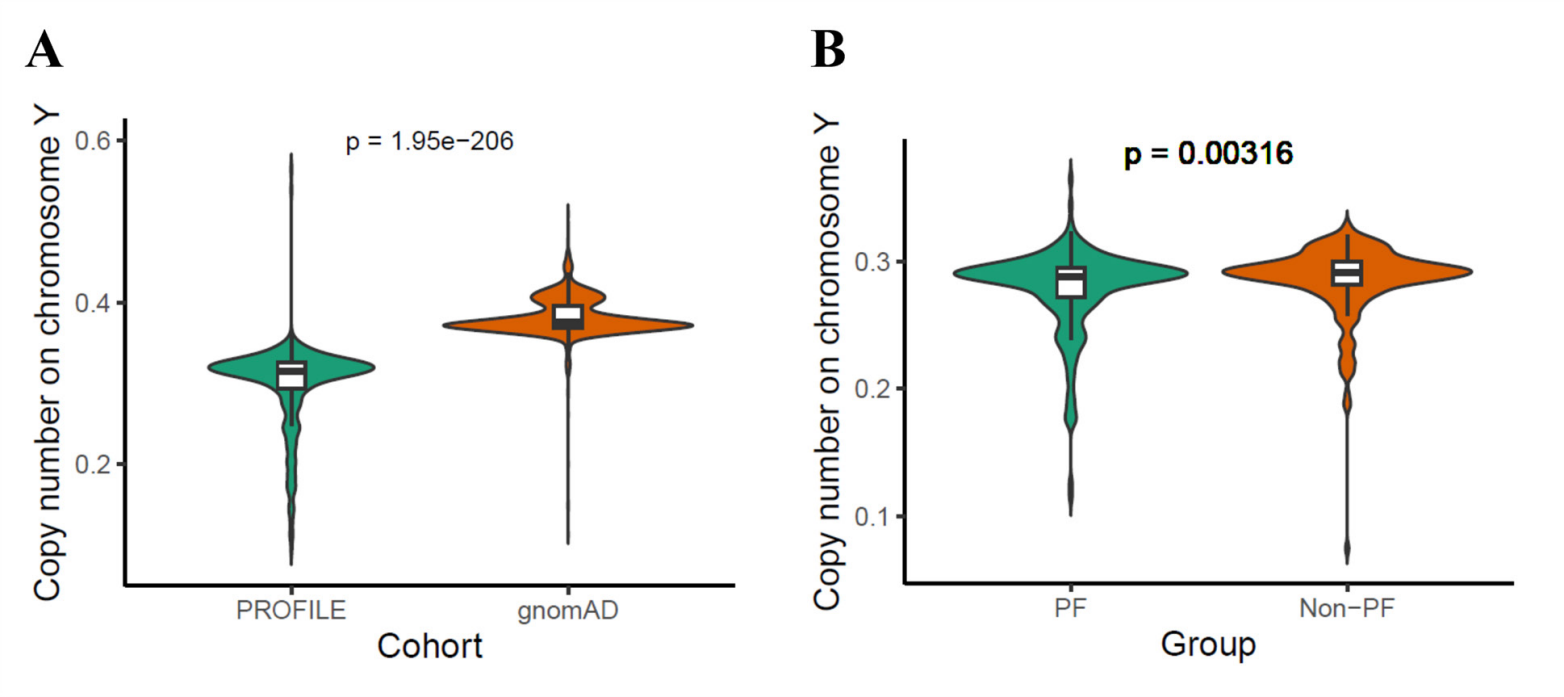
mLOY in case and control cohort. (**A**) Copy number on chromosome Y for male PF case from PROFILE and non-PF control patients from gnomAD cohort. Copy number on chromosome Y is defined as mean read depth on MSY divided by mean read depth on chromosome 20. (**B**) Copy number on chromosome Y for male PF case and non-PF control patients from GE100KGP cohort. Copy number on chromosome Y is defined as mean read depth on MSY divided by mean read depth on 22 autosomes. The Wilcoxon test was used for testing the difference between two groups. mLOY, mosaic loss of chromosome Y; MSY, male-specific region Y; PF, pulmonary fibrosis.

In total, 387 male patients in PROFILE case cohort (age: 70.6±8.3) and 232 male patients in GE100KGP case cohort (age: 67.1±13.5) as well as 2216 male patients in gnomAD control cohort and 232 male patients in GE100KGP control cohort were included (online supplemental Figure S1). The copy number on chromosome Y was significantly lower in the PROFILE case cohort (median: 0.315) than the gnomAD control cohort (median: 0.375; p=1.95e−206) (figure 1A); however, the differences observed might be partly attributable to the differences in sequencing platform and variant calling pipeline from the two cohorts (online supplemental Table S1). To account for age-related effects, a propensity matched non-PF control cohort was generated from the GE100KGP dataset, and the copy number on chromosome Y in GE100KGP case cohort (median: 0.288) was marginally but significantly lower than that in GE100KGP control cohort (median: 0.291; p=0.00316) (figure 1B). Overall, lower copy number on chromosome Y was observed in PF patients compared with non-PF patients in both cohort analyses, indicating an association between mLOY and PF. However, age was highly negatively correlated with copy number on chromosome Y for both PROFILE cohort (Spearman: R=−0.24, p=1.2e−6) and GE100KGP cohort (Spearman: R=−0.38, p=2.3e−9).

### mLOY is related to age and telomere length but not PF severity or disease progression

**Figure 2:**
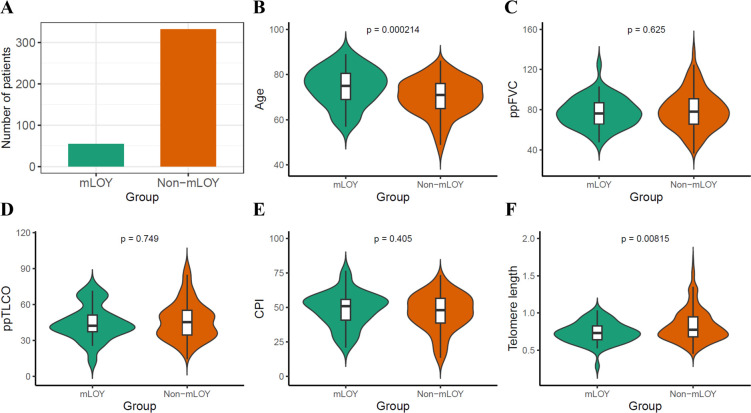
Compare the age, lung function and telomere length between mLOY and non-mLOY group male PF patients from the PROFILE cohort. (**A**) Number of PF patients of mLOY and non-mLOY group (**B**) Age (**C**) ppFVC (**D**) ppTLCO (**E**) CPI (**F**) Telomere length. The Wilcoxon test was used for testing the difference between two groups. CPI, composite physiologic index; mLOY, mosaic loss of chromosome Y; PF, pulmonary fibrosis; ppFVC, percent of predicted forced vital capacity; ppTLCO, percent of predicted transfer factor of the lung for carbon monoxide.

**Figure 3:**
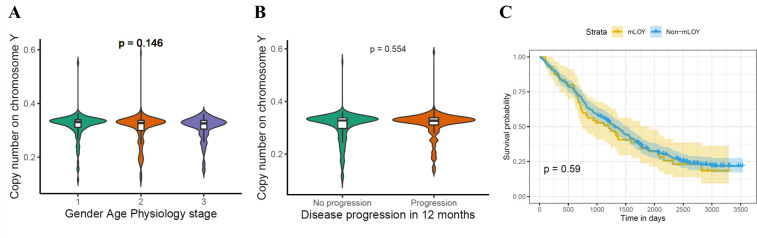
Correlation of mLOY for male PF patients from the PROFILE cohort with disease severity. (**A**) Gender-Age-Physiology (GAP) stage. The Kruskal-Wallis test was used for testing the difference among the three groups. (**B**) Progression in 12 months. The Wilcoxon test was used for testing the difference between two groups. Copy number on chromosome Y is defined as mean read depth on MSY divided by mean read depth on 22 autosomes. (**C**). Kaplan-Meier plot for survival analysis. The log-rank test was used for the comparison of two survival curves. mLOY, mosaic loss of chromosome Y; MSY, male-specific region Y; PF, pulmonary fibrosis.

To investigate whether mLOY is directly involved in PF pathogenesis and the relationship between mLOY and key demographic features, we defined a threshold for substantial mLOY as a copy number of <0.2565 and non-mLOY as a copy number of ≥0.2565. Patients with substantial mLOY were significantly older (75 vs 71; p=0.000214) and had significantly shorter telomeres (0.728 vs 0.775; p=0.00815) than patients without mLOY. However, no significant difference in ppFVC (p=0.625), and ppTLCO (p=0.749) was found (figure 2). Consistently, a linear regression analysis adjusting for age and smoking history found significant positive association between telomere length and copy number on chromosome Y (p=0.0185) but no association between the lung function measurements and copy number on chromosome Y: ppFVC (p=0.862), and ppTLCO (p=0.653) (online supplemental Table S2). Furthermore, no significant difference was found in the copy number on chromosome Y between patients that did progress in 12 months (N=179) and patients that did not progress in 12 months (N=208; p=0.554) (figure 3). To further explore the data using survival analysis, the log-rank test for Kaplan-Meier survival curves showed no significant difference in survival between mLOY and non-mLOY groups (p=0.59, figure 3C). The multivariate analysis using Cox proportional hazards model suggested that the classification of mLOY and non-mLOY group is not significantly associated with patients’ risk of death (p=0.561) and older patients are significantly associated with higher risk of death (p=0.00175, HR=1.02) and greater ppFVC is significantly associated with lower risk of death (p<2e−16, HR=0.963, (online supplemental Table S3). Stratification of mLOY by the risk alleles from SAC genes associated with IPF did not enrich patients with mLOY, suggesting that the causal mechanisms for SAC genes were not directly related to mLOY (online supplemental Figure S2).

### Single-cell analysis

**Figure 4:**
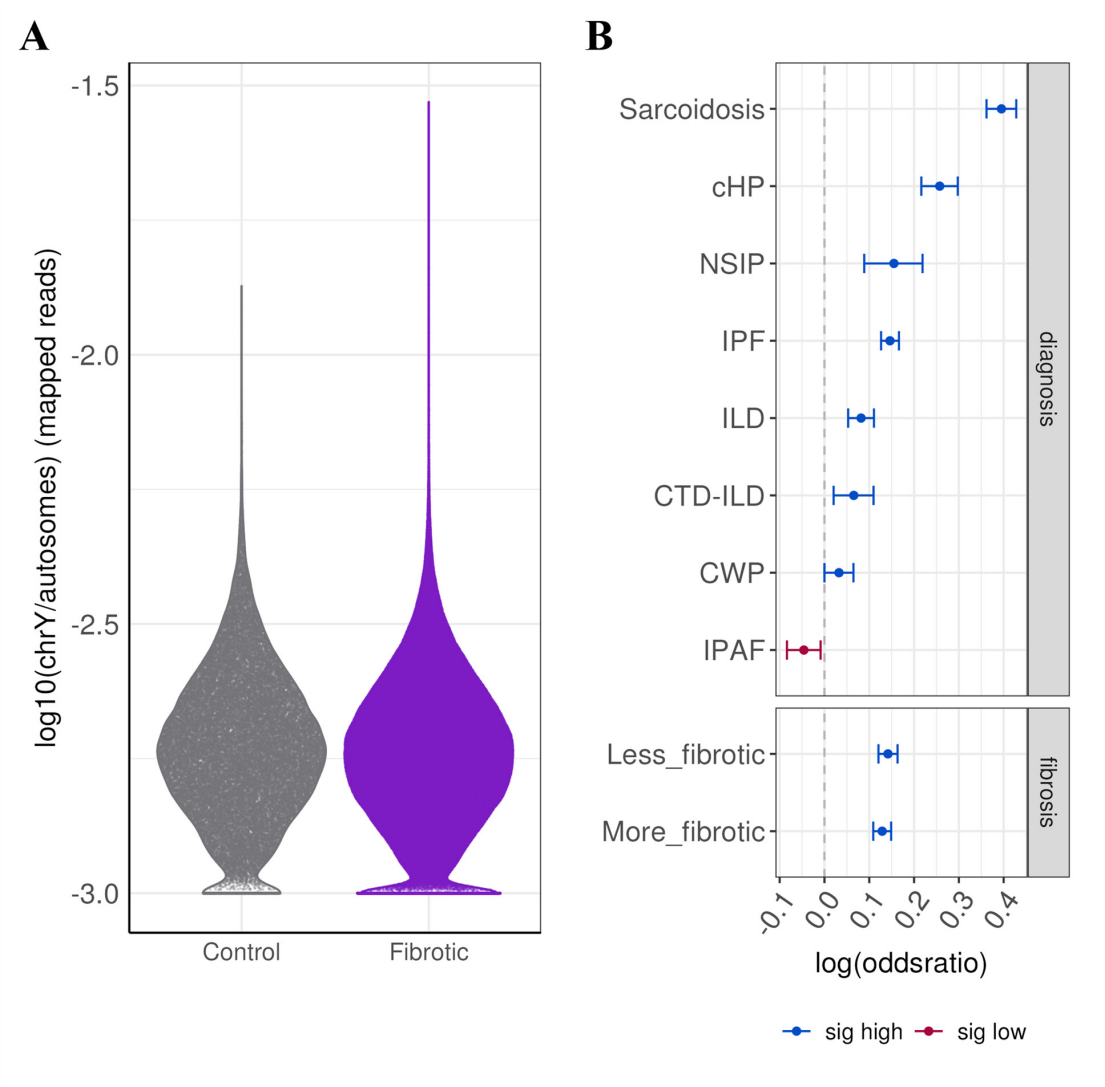
Increased mLOY in interstitial lung diseases. (**A**) Distribution of chrY/autosomes ratio across cells from lung samples collapsed by control aged samples and fibrotic samples. Plot is scaled by width. (**B**) Association between mLOY probability and disease according to ILD type and fibrosis status. Significant results (FDR-adjusted p<0.05, logistic regression) are coloured in blue (increased probability of mLOY) or red (decreased probability of mLOY). Values represent OR and 95% CI. cHP, chronic hypersensitivity pneumonitis; CTD-ILD, connective tissue disease-associated interstitial lung disease; CWP, coal worker’s pneumoconiosis; FDR, false discovery rate; ILD, interstitial lung disease; IPAF, interstitial pneumonia with autoimmune features; IPF, idiopathic pulmonary fibrosis; mLOY, mosaic loss of chromosome Y; NSIP, non-specific interstitial pneumonia.

To understand whether mLOY in structural cells is associated with PF, mLOY was assessed using lung single-cell transcriptomic data from 44 males with PF and 27 control males. Cells from PF samples have a higher probability of losing chromosome Y compared with controls, as evidenced by the absence of mapped read to chromosome Y (figure 4, online supplemental Figure S3A). Patients diagnosed with IPF show significantly higher probability of mLOY compared with controls (figure 4B, online supplemental Figure S3B). This finding extends to other types of ILDs, including sarcoidosis, cHP, NSIP, CWP and CTD-ILD, all of which exhibit an increase in probability of mLOY compared with controls (figure 4B, online supplemental Figure S3B). To determine whether specific cell types are more susceptible to mLOY, the probability of mLOY occurring in a cell-type specific manner was assessed. At the lineage cell level, immune, mesenchymal and endothelial cells exhibited a significantly higher probability for mLOY in fibrotic tissue compared with controls, whereas epithelial cells showed a decreased probability of mLOY (online supplemental Figure S4A). Cell-type specific mLOY was primarily observed in immune cells and included plasma cells, mast cells, dendritic cells, CD4+cells, monocyte-derived macrophages and alveolar macrophages (online supplemental Figure S4A). Among PF patients who had paired samples from more and less fibrotic regions of the lung, alveolar macrophages from more fibrotic regions had a higher probability of mLOY compared with samples from less fibrotic regions (online supplemental Figure S4B). mLOY in other immune cells showed a similar trend (online supplemental Figure S4B). While epithelial cells showed lower probability of mLOY, AT2 cells had significantly higher probability for mLOY in fibrotic tissue (online supplemental Figure S4A).

To assess whether mLOY is associated with changes in expression signature, differential expression analysis was carried out using cell-type agnostic and cell-type specific approaches between mLOY cells and the top 25th percentile of cells based on their reads mapped to chrY and reads mapped to autosomes ratio. Differential expression by cell type showed that expression changes associated with mLOY were predominately observed in immune cells, including monocyte-derived macrophages, inflammatory monocytes, CD4+cells, dendritic cells (moDC) and proliferating immune cells (online supplemental Figure S5A). However, a high number of differentially expressed genes were also observed in epithelial cells (ciliated cells, MUC5B+secretory cells) and endothelial cells (systemic venous, venule). Expression changes were cell-type specific and showed little overlap across cell types (online supplemental Figure S5B). GSEA shows that in immune cells, mLOY was associated with activation of genes of metabolic pathways (online supplemental Figure S5C). To assess cell-common gene expression changes that are associated with mLOY, differential expression was performed using a cell-type agnostic approach. A total of 1173 differentially expressed genes were identified (online supplemental Figure S5A, FDR adjusted p<0.05 and |std. regression coefficient|>0.05). Enrichment analysis reveals activation of genes related to the lysosomal and phagosome, activation of immune-related signalling pathways and apoptosis (online supplemental Figure S5C). The single-cell transcriptomic data support the role of specific types of immune cells in mLOY in PF patients.

### mLOY is not on the causal pathway for IPF but short telomeres may be on the causal pathway for mLOY

**Figure 5:**
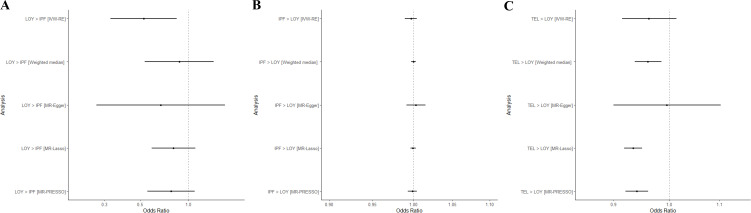
Forest plot from Mendelian randomisation (MR) analysis for the causal association. (**A**) mLOY and IPF (**B**) IPF and mLOY (**C**) Telomere length and mLOY. IPF, idiopathic pulmonary fibrosis; mLOY, mosaic loss of chromosome Y; TEL, telomere length.

To understand the causal relationship between mLOY, IPF and telomere length, Mendelian randomisation was performed. For Mendelian randomisation between mLOY and IPF, a negative effect of mLOY on IPF was identified (IVW-RE, OR=0.526; 95% CI 0.328 to 0.845; p=0.008) through the IVW-RE method. However, high heterogeneity suggested that horizontal pleiotropic effects were likely to be present (Q=357.51, p<1.00×10^−4^, I^2^=62.50%). No effects were detected after correcting for pleiotropy (figure 5A) and removal of likely pleiotropic SNPs using MR-Lasso resulted in greatly reduced heterogeneity (Q=109.02, p=0.71, I^2^=0.00%). No causal effect of IPF on mLOY was identified (IVW-RE, OR=0.997; 95% CI 0.989 to 1.004; p=0.377) (figure 5B). Results indicated significant heterogeneity was present in the IWV-RE analysis of IPF on mLOY (Q=213.95, p<1.00×10^−4^, I^2^=91.60%), however, outcomes were consistent across IVW-RE and pleiotropy-robust analyses (figure 5B). As a negative control, no causal effect of mLOY on ovarian cancer was found for all five methods (IVW-RE, OR=1.047; 95% CI 0.865 to 1.269; p=0.634) (online supplemental Figure S6A) and as a positive control, causal effect of mLOY on prostate cancer was identified from all five methods (IVW-RE, OR=1.461; 95% CI 1.247 to 1.711; p=2.56e−6) (online supplemental Figure S6B), which is consistent with the effects identified in a previous study.^10^ Three out of five methods indicated a negative causal effect of telomere length on mLOY, although the result from the IVW-RE method was not significant (IVW-RE, OR=0.963; 95% CI 0.915 to 1.014; p=0.153) (figure 5C). Heterogeneity was also indicated to be high in the IVW-RE analysis of telomere length on mLOY (Q=202.54, p<1.00×10^−4^, I^2^=39.80%) but was greatly reduced following removal of likely pleiotropic variants with MR-Lasso (Q=110.13, p<0.59, I^2^=0.00%). Leave-one-out sensitivity analyses also identified significant effects of shorter telomere length on mLOY when either rs16978028 or rs7705526 is removed (online supplemental Figure S7).

## Discussion

These data demonstrate that although mLOY is increased in blood cells in male patients with PF compared with non-PF patients the effect is modest. Furthermore, there was no association between blood mLOY and disease progression and outcome. Single-cell transcriptomic analysis of lung tissue from patients with a variety of fibrotic diseases identified that mLOY in immune cells was associated with fibrotic diseases, and there was an association with increased fibrosis within samples. However, bidirectional Mendelian randomisation did not find any causal associations between IPF and mLOY, although there was some evidence that shorter telomere length was on the causal pathway for mLOY.

mLOY and PF share several risk factors such as age, smoking and telomere shortening. It is well known that a positive correlation exists between the prevalence of mLOY and age in the general male population.^10^ The subtle difference in mLOY between the PF case cohort and age-matched non-PF cohort suggests that the effect of age is driving mLOY in PF. Previous Mendelian randomisation analysis has demonstrated that telomere shortening is on the causal pathway for IPF but not COPD.^36^ The indication that telomere length is on the causal pathway of both IPF and mLOY supports a model where telomere length is on the causal pathway for both IPF and mLOY which may explain some comorbidities more directly attributable to mLOY.^13^

It has been widely recognised that a variety of innate and adaptive immune cells interacting with other types of cells could be involved throughout the development of IPF, especially after lung injury and infection.^37^ A feed-forward mechanism suggests that activation of immune cells and fibroblasts releases proinflammatory cytokines and chemokines to strengthen the existing activation.^38^ The direct causal relationship between immunophenotypes and IPF risk has been confirmed by a Mendelian randomisation analysis.^39^ A dysfunctional immune system with other pro-fibrotic pathways may contribute to the long-term unresolved inflammation leading to the formation of fibrosis, while different types of cells might play opposite roles, suggested by studies on IPF and mouse models.^40^ Our study identifies three types of immune cells in which mLOY is related to fibrosis disease and fibrosis severity: CD4+cells, monocyte-derived macrophages and alveolar macrophages. The number of CD4+cells is reported to increase in IPF lungs, suggesting its functional relevance to IPF.^41^ CD4+ T lymphocytes have been found to be associated with increased level of mLOY in prostate cancer patients whereas mLOY is enriched in NK cells for Alzheimer’s disease patients, suggesting that the association of CD4+cells with mLOY exhibits disease specific features.^42^ Macrophages represent the most common immune cells in the human lung, and alveolar macrophages are one of the two major types of pulmonary macrophages.^43^ Significantly reduced efferocytosis in alveolar macrophages and impaired mitochondrial homeostasis in alveolar macrophages observed in IPF patients link alveolar macrophages to the pathogenesis of IPF. Alveolar macrophages can also impact normal and IPF fibroblasts to differing extents through cell-cell communication.^44^ Pro-inflammatory cytokine expressed classically activated (M1) and anti-inflammatory cytokine expressed alternatively activated (M2) phenotypes exert polarised effects on the responses to the injury.^43^ Persistent lung injury recruits the monocyte-derived macrophage cells and transits macrophages into M2 phenotype under the regulation of TGF-β signalling pathway.^45 46^ Our findings suggest that ILD subtype may play a role in mLOY increase. Fibrotic ILD subtypes such as sarcoidosis,^47^ cHP^48^ and NSIP^49^ exhibit a higher pulmonary immune burden with denser infiltration and activation of macrophages, CD4+T cells and other leucocytes. This heightened proliferative and inflammatory stress likely renders these subtypes more susceptible to mLOY in immune populations. The findings that these important immune cells experimentally proved to be involved in the IPF disease molecular mechanism are profoundly impacted by mLOY highlight the potential role of mLOY in the pathogenesis of IPF. This will provide valuable information for the appropriate design and use of treatments for IPF patients, such as immunosuppressive medications.

This study has a number of strengths, including the use of propensity matching, analysis of single cells and the undertaking of Mendelian randomisation. For each type of analysis, we either performed the analysis on multiple case–control cohorts or conducted sensitivity analyses to generate the most robust results. However, there were several limitations. We used the copy number on chromosome Y from whole genome sequencing as a proxy for mLOY for each patient, and mLOY status could be confirmed using the experimental approaches such as fluorescence in situ hybridisation. A recent study using different techniques to measure mLOY shows that men with mLOY have higher IPF risk and that mLOY-positive lung leucocytes upregulate profibrotic TGF-β signalling.^50^ While our Mendelian randomisation does not support mLOY as a causal driver of IPF, our results align in suggesting that telomere shortening predisposes to mLOY, which may then amplify profibrotic activity in affected immune cells. This supports a model where telomere dysfunction initiates risk, and mLOY may modify disease biology in a subset of patients. The study is limited by the availability of the specific datasets. For the genetic analysis, the GE100KGP cohort is a disease-enriched cohort with which the analysis might overestimate the strength of mLOY in the age-matched control. For future work, a case–control cohort study to include the PF cases and age-matched healthy controls is needed to validate the findings.

In conclusion, whole genome sequencing data analysis, single-cell transcriptomic analysis and Mendelian randomisation analysis show that mLOY is related to PF but mLOY is not on the causal pathway of IPF. We propose that telomere shortening may be causing both mLOY and IPF through different molecular mechanisms. Further experimental validation should be performed to test this hypothesis.

## Supplementary material

10.1136/bmjresp-2025-003846 online supplemental file 1

## Data Availability

Data are available on reasonable request.
