# Supplementary material for: Assessment of mosaic loss of chromosome Y in pulmonary fibrosis reveals limited association with susceptibility or disease severity
Source: BMJ Open Respir Res. 2026 Feb 10;13(1):e003846. doi: 10.1136/bmjresp-2025-003846 (PMC12911719; doi:10.1136/bmjresp-2025-003846)
Supplement: online supplemental file 1 [file bmjresp-13-1-s001.docx]

**Supplementary material**

**Figure S1. Age distribution for male PF patients.**

(A) PROFILE cohort (B) GE100KGP cohort.


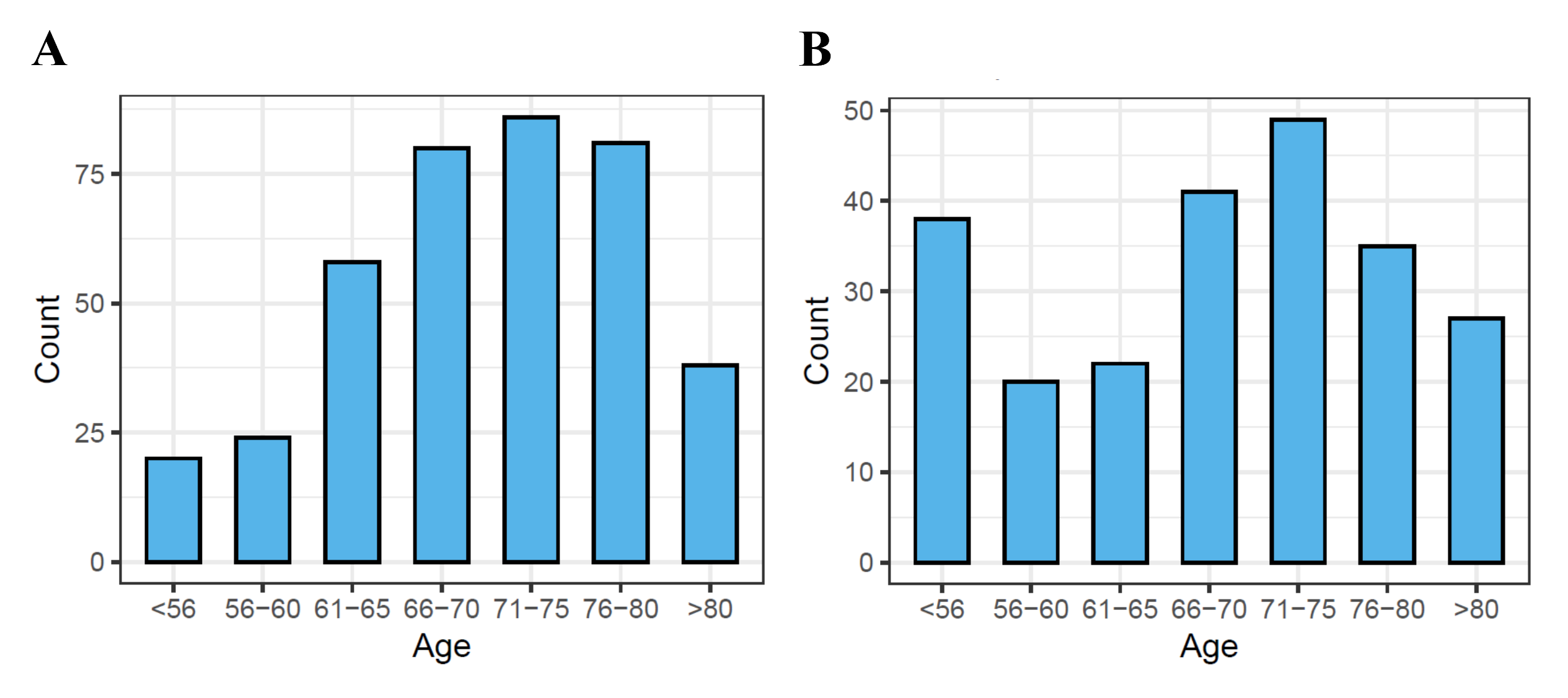


**Figure S2. Association of mLOY and risk alleles from four SAC genes for PF male patients from PROFILE cohort.**

(A) Number of patients without risk alleles and with risk alleles. (B) Fisher's exact test for relationship between mLOY and risk alleles.


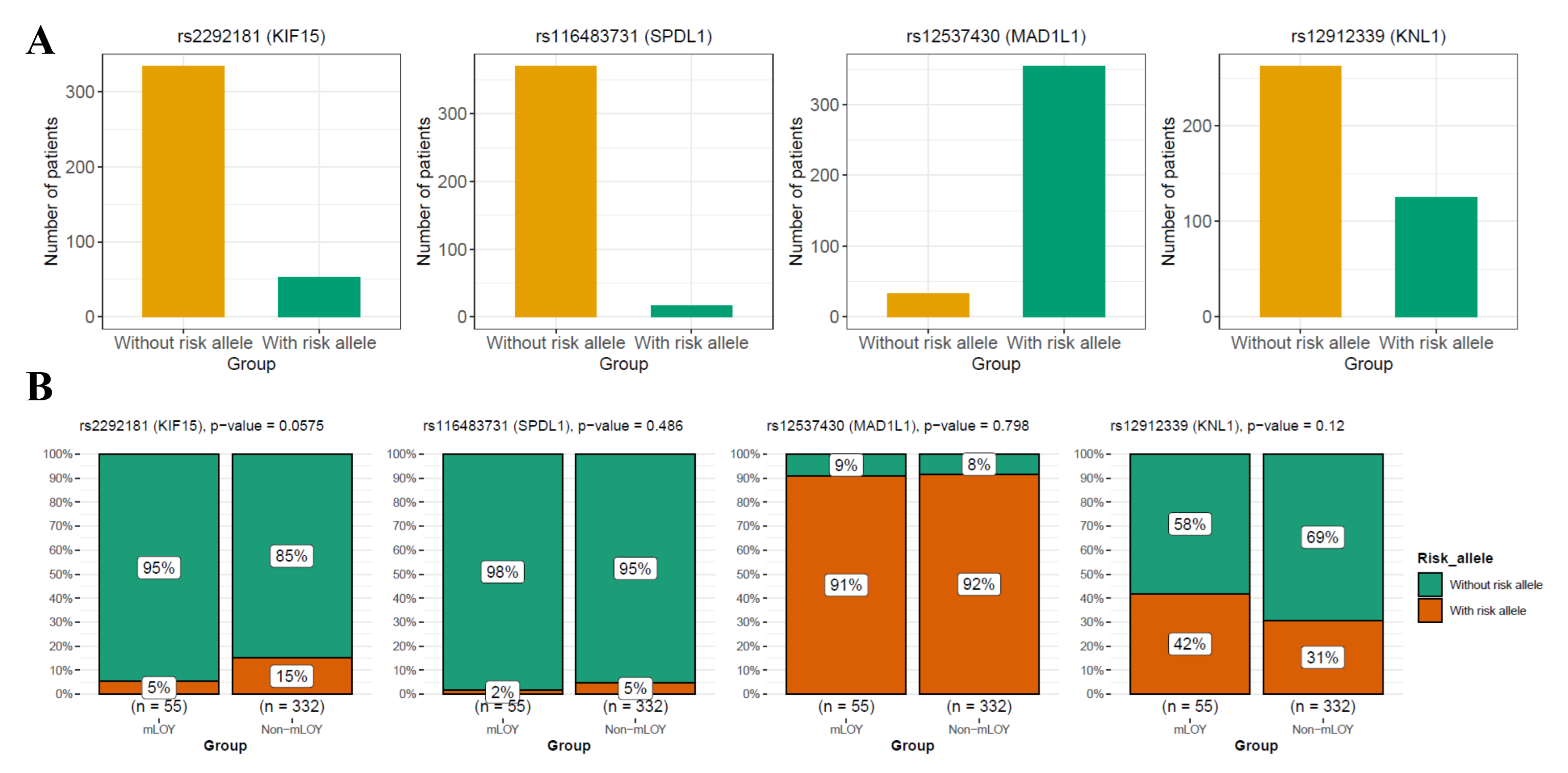


**Figure S3. mLOY across samples and disease types.**

Percent of cells showing mLOY across samples coloured by (A) disease status and (B) diagnosis. cHP, chronic hypersensitivity pneumonitis; CTD-ILD, connective tissue disease-associated interstitial lung disease; ILD, interstitial lung disease; IPF, idiopathic pulmonary fibrosis; CWP, coal worker’s pneumoconiosis; IPAF, interstitial pneumonia with autoimmune features; NSIP, nonspecific interstitial pneumonia.


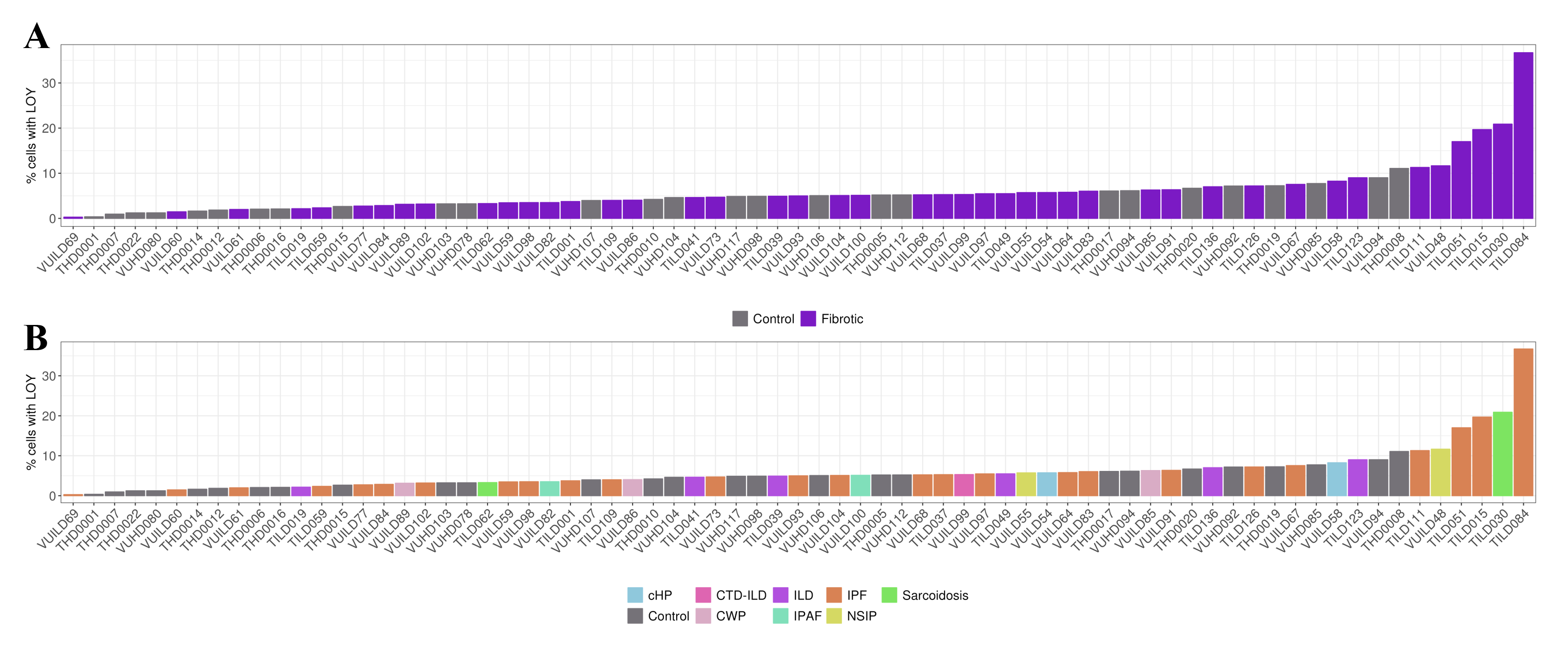


**Figure S4. mLOY increases with fibrosis primarily in immune cells.**

(A) Association between mLOY probability and disease according to disease or cell-type. Shape corresponds to less fibrotic (circle) or more fibrotic (triangle) samples. (B) Association between mLOY probability and disease progression comparing more fibrotic samples to less fibrotic samples. Significant results (FDR adjusted p-value < 0.05, logistic regression) are coloured by either blue or red, highlighting higher or lower probability of mLOY with fibrosis, respectively. Values represent odds ratio and 95% CI.


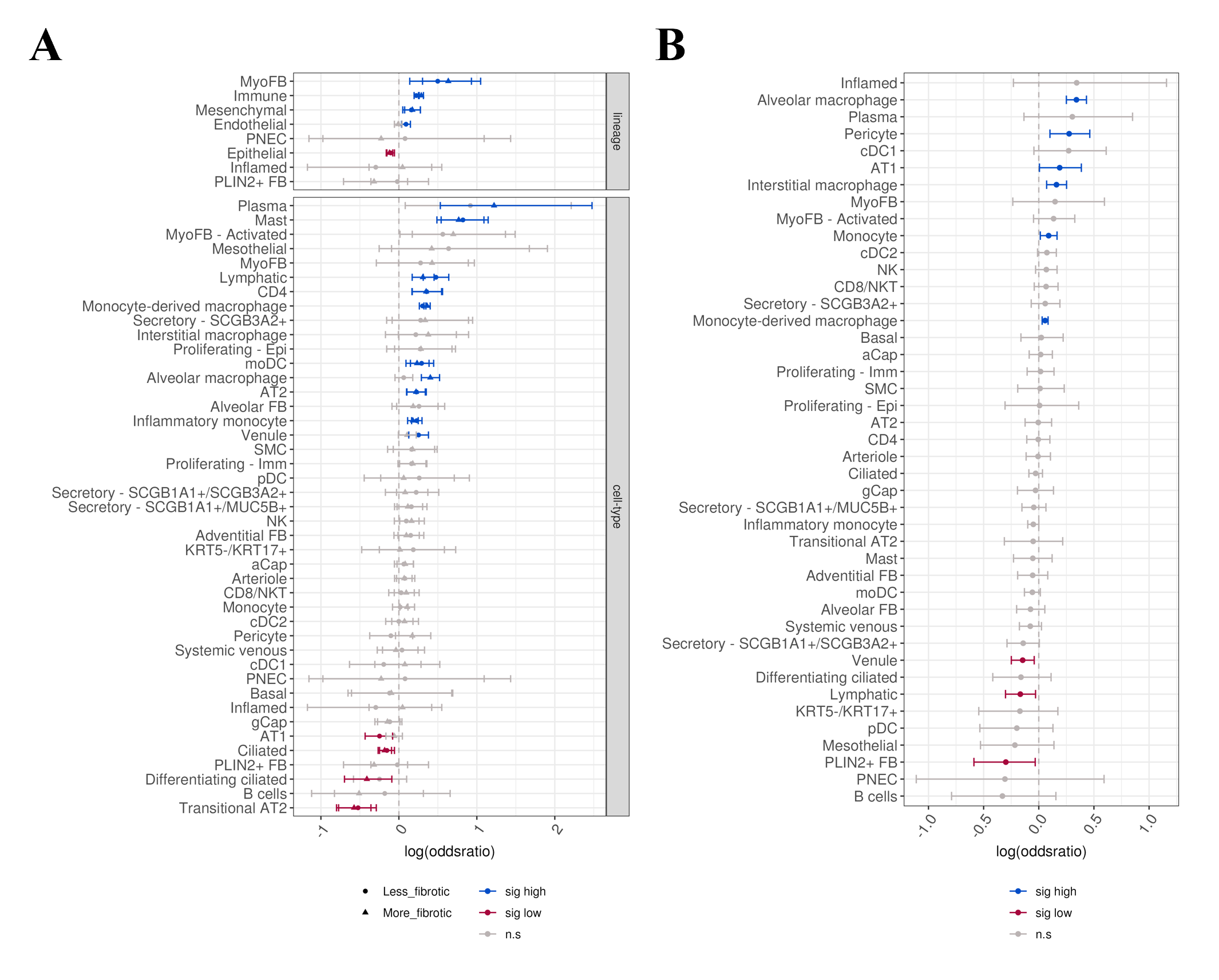


**Figure S5. Differential expression between cells lacking chromosome Y and top 25% chrY/autosomes ratio.**

(A) Number of differentially expressed genes between cells with mLOY and the top 25% of cells for chrY/autosomes ratio (B) Upset plot showing the intersection between differentially expressed genes across cell types (C) GSEA results for each cell types.


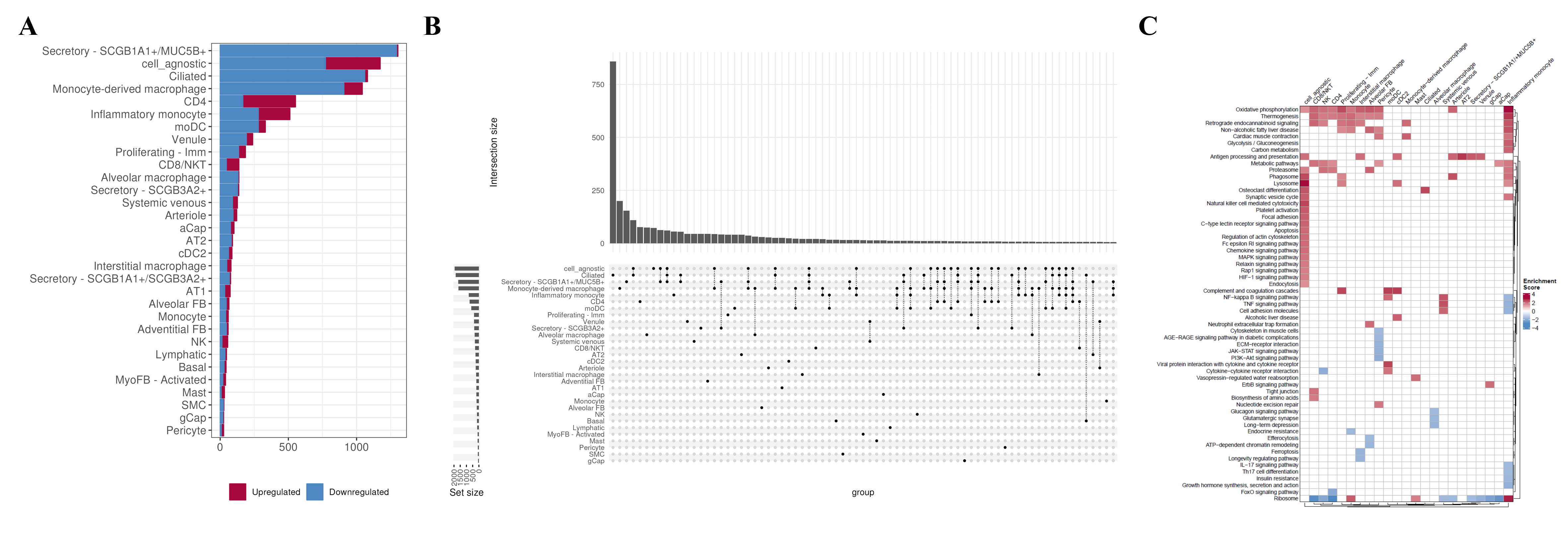


**Figure S6. Forest plot from Mendelian randomization analysis for the causal association.**

(A) mLOY and ovarian cancer (negative control) (B) mLOY and prostate cancer (positive control).


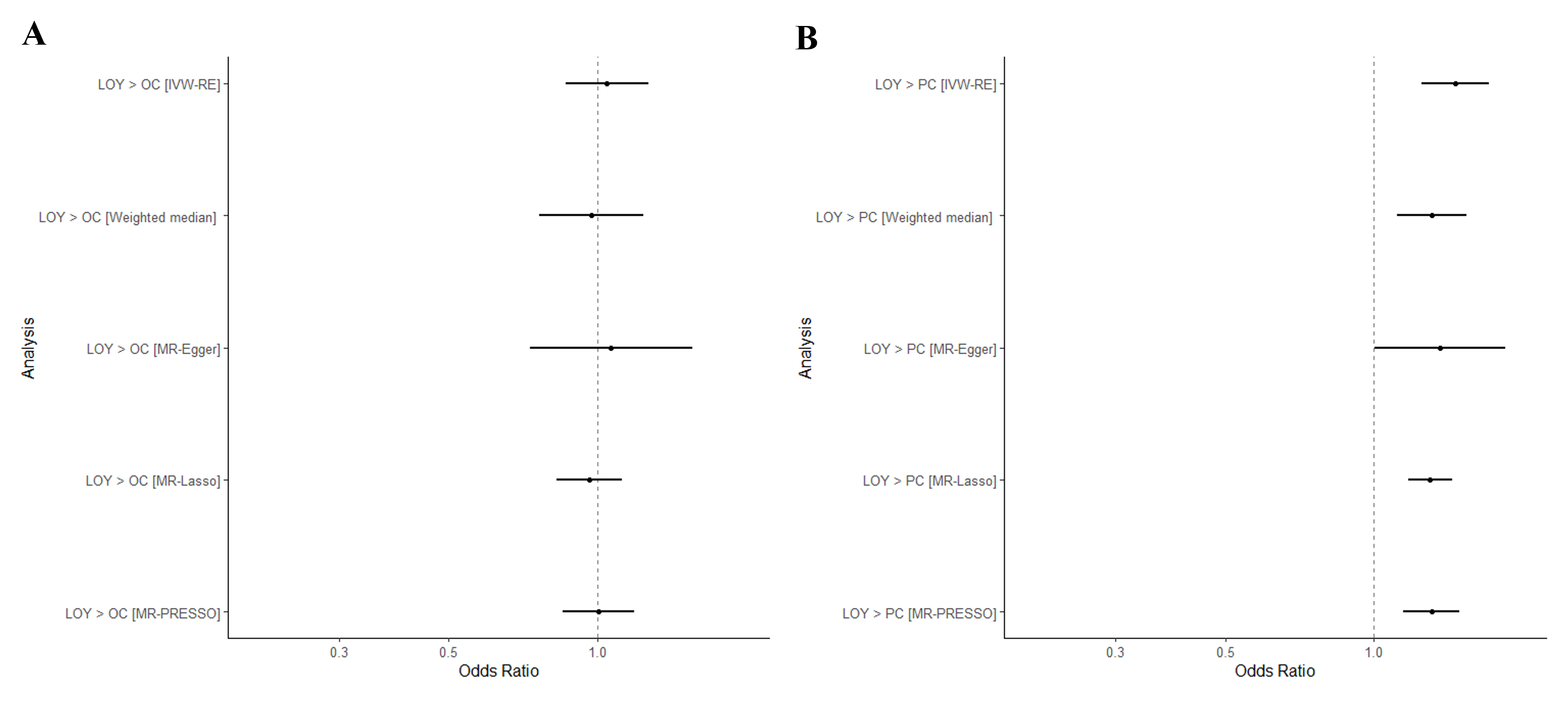


**Figure S7. Results of Mendelian randomization analysis for the causal association between telomere length and mLOY.**

(A) Scatter plot (B) Leave one out plot (C) Single-SNP estimates forest plot.


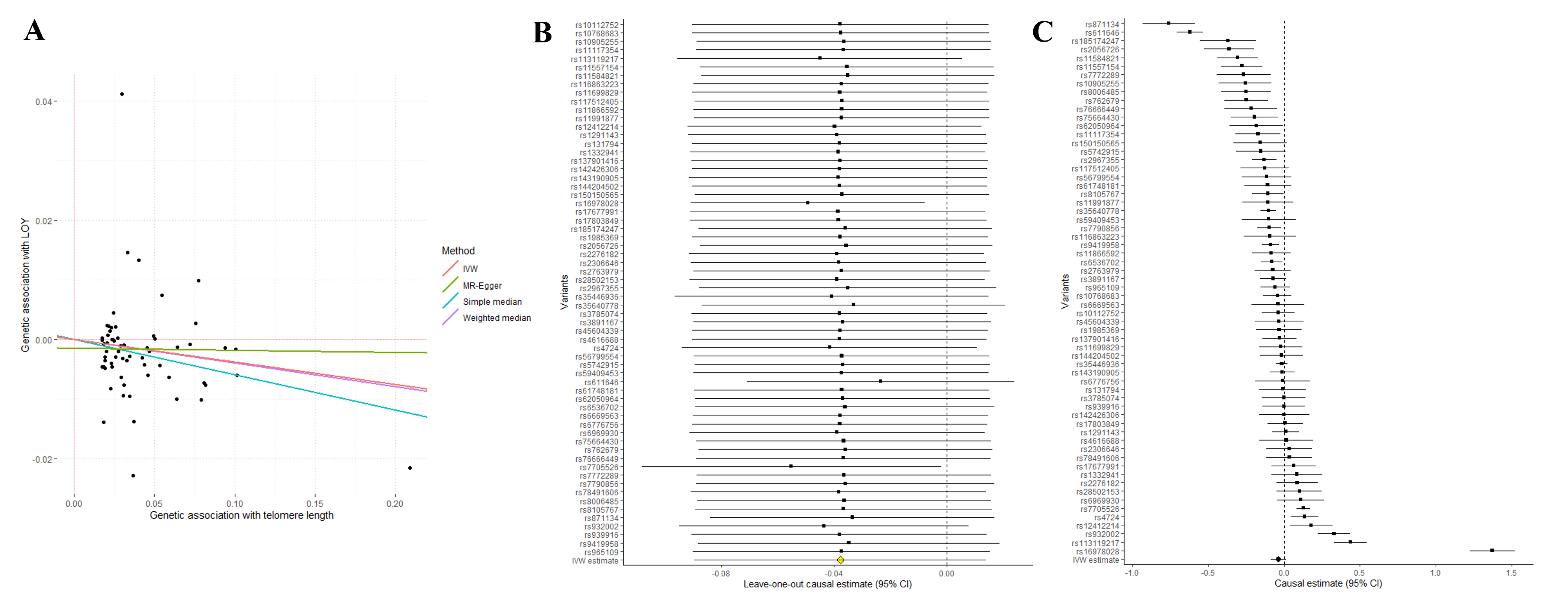


**Table S1. Comparison of technical details between PROFILE and gnomAD cohort.**

|  | PROFILE | gnomAD |
| --- | --- | --- |
| Sequencing platform | NovaSeq 6000 | Illumina HiSeq X/NovaSeq 6000 |
| Variant calling pipeline | Illumina DRAGEN Bio-IT Platform Germline Pipeline v3.0.7 | GATK Best Practices pipeline/Hail |
| Mean read depth for chromosome Y (SD) | 13.3 (2.9) | 12.5 (1.5) |
| Mean read depth for chromosome 20 (SD) | 44.3 (5.7) | 32.9 (4) |

**Table S2. A linear regression analysis adjusting for age and smoking history for lung function measurements and telomere length of male PF patients from PROFILE cohort with copy number on chromosome Y.**

| Independent variable | Estimate | Std. Error | t value | Pr(>\|t\|) |
| --- | --- | --- | --- | --- |
| ppFVC | 2.49E-05 | 1.43E-04 | 0.173 | 0.862 |
| ppTLCO | 7.92E-05 | 1.76E-04 | 0.45 | 0.653 |
| CPI | -8.66E-05 | 2.09E-04 | -0.414 | 0.679 |
| Telomere length | 0.0294001 | 0.0124302 | 2.365 | 0.0185 |

**Table S3. Univariate and multivariate analysis using Cox proportional hazards model.**

|  | Univariate | | Multivariate | |
| --- | --- | --- | --- | --- |
|  | Hazard ratio (95% confidence interval) | P-value | Hazard ratio (95% confidence interval) | P-value |
| Group (mLOY) | 1.09 (0.786-1.52) | 0.591 | 0.9 (0.631-1.28) | 0.561 |
| Age | 1.02 (1-1.03) | 0.0173 | 1.02 (1.01-1.04) | 0.00175 |
| ppFVC | 0.965 (0.957-0.972) | 6.63E-21 | 0.963 (0.956-0.971) | < 2e-16 |
